# Comparison Between the Fecal Bacterial Microbiota of Healthy and Diarrheic Captive Musk Deer

**DOI:** 10.3389/fmicb.2018.00300

**Published:** 2018-03-02

**Authors:** Yimeng Li, Xiaolong Hu, Shuang Yang, Juntong Zhou, Lei Qi, Xiaoning Sun, Mengyuan Fan, Shanghua Xu, Muha Cha, Meishan Zhang, Shaobi Lin, Shuqiang Liu, Defu Hu

**Affiliations:** ^1^College of Nature Conservation, Beijing Forestry University, Beijing, China; ^2^College of Animal Science and Technology, Jiangxi Agricultural University, Nanchang, China; ^3^Research Department, Zhangzhou Pien Tze Huang Pharmaceutical Co., Ltd., Zhangzhou, China

**Keywords:** bacterial microbiota, diarrheic, musk deer, 16S-rRNA gene sequencing, functional prediction

## Abstract

Diarrhea constitutes one of the most common diseases affecting the survival of captive musk deer and is usually caused by an imbalance in intestinal microbiota. Currently, research regarding the structure and function of intestinal microbiota in diarrheic musk deer is lacking. Therefore, in the present study, high-throughput 16S-rRNA gene sequencing was used to analyze the intestinal microbiota in feces of healthy captive musk deer (HMD) (*n* = 8) and musk deer with mild (MMD) (*n* = 8), and severe (*n* = 5) (SMD) diarrhea to compare the difference in intestinal microbiota of musk deer under various physiological conditions. The results showed that the diversity of HMD fecal microbiota was significantly higher than that of the two diarrhea samples. β Diversity results indicated that there were extremely significant differences in bacterial communities between the HMD sample and the MMD and SMD samples. However, no significant difference was found between the two diarrhea samples. LefSe analysis showed that the degree of intestinal physiological dysfunction in musk deer was correlated with the types of major pathogens. The main pathogen in the MMD group is *Escherichia–Shigella*, whereas *Fusobacterium* is the main pathogen in the SMD group. PICRUSt functional profile prediction indicated that the intestinal microbiota disorder could also lead to changes in the abundance of genes in metabolic pathways of the immune system. Altogether, this study provides a theoretical basis for the exploration of treatments for diarrhea in captive musk deer, which is of considerable significance to the implementation of the musk deer release into the wild program.

## Introduction

Since 1958, China has been experimenting with forest musk deer (*Moschus berezovskii*) farming and has achieved significant progress across many aspects over the past decades (Wu and Wang, 2006; He et al., 2014). The initial goal of farming musk deer is to reduce the pressure on wild musk deer resources for obtaining valuable musk. With the sharp reduction of the wild musk deer population, the captive-bred musk deer population will also constitute an important source of reintroduction (Wang et al., 2015). However, the captive bred forest musk deer population has long been affected by factors such as unstable health and high incidence of disease, which seriously restricts population development (Li et al., 2012; He et al., 2014; Wei Y.T. et al., 2016; Wei Z. et al., 2016). Among these diseases, diarrhea represents one of the most common diseases in captive-bred forest musk deer. Notably, severe cases can lead to death, and in some musk deer farms, the mortality of diarrhea reaches 30% (Yan et al., 2016). In particular, diarrhea can be caused by issues such as forage replacement in spring and fall, the rainy season in summer, and hygiene conditions of pens. Therefore, in recent years, diarrhea has become a focus of disease prevention and control for captive-bred musk deer, although the relevant research remains lacking.

Numerous studies have shown that the occurrence of intestinal diseases in mammals is closely related to the structure of intestinal microbiota (Frank et al., 2007; Packey and Sartor, 2009; Craven et al., 2012). Intestinal microbiota constitute the main part of microorganisms inside host. It has been demonstrated that intestinal microbiota are important for the health and nutrition of hosts by aiding colonic fermentation (Koboziev et al., 2014), stimulating the immune system (Mazmanian et al., 2005), defensing pathogens invasion (Kamada et al., 2012), and improving energy acquisition (Turnbaugh et al., 2006). Accordingly, the intestinal microbiota is crucial for the normal physiological homeostasis of its host. When the normal microbiota is affected by exogenous harmful microorganisms, its self-balance will be broken, which may lead to digestive disorders or even induce disease of the host. In particular, regardless of the cause of diarrhea, the gut microbiota often shows a serious imbalance (Wang, 2015). Recent studies have shown that the types of microbes in the intestine are abundant and can be affected by the external environment (Hu et al., 2017; Li et al., 2017). However, the characteristics of intestinal microbiota in musk deer with diarrhea remain unclear. Therefore, this study aimed to utilize high-throughput 16S-rRNA gene sequencing to comprehensively analyze and compare the composition and structure of fecal microbiota of healthy forest musk deer and deer with diarrhea, with the goal of providing a scientific basis for the prevention and control of diarrhea of musk deer, thereby improving the health of the captive musk deer population.

## Materials and Methods

### Ethics Statement

This study was carried out in accordance with the recommendations of the Institution of Animal Care and the Ethics Committee of Beijing Forestry University. The protocol was approved by the Ethics Committee of Beijing Forestry University. The collection of captive musk deer stool samples was approved by the Pien Tze Huang Forest Musk Deer Breeding Center.

### Sample Collection

Experimental forest musk deer were obtained from the Pien Tze Huang Forest Musk Deer Breeding Center, Shaanxi. Ear tags were used to identify individual musk deer, and those that had received anthelmintic or antibiotic treatments in the past 2 months were excluded. The musk deer are housed alone in each enclosure at night to allow for feces to be collected from specific individuals. 4–6-year-old adult forest musk deer were selected, including 8 healthy musk deer (HMD) (Numbers H1–H8), 8 musk deer with mild diarrhea (MMD) (Numbers M1–M8), and 5 musk deer with severe diarrhea (SMD) (Numbers S1–S5). A total of 21 fecal samples were collected. The identification criteria of HMD deer feces were as follows: droppings exhibited a clear pellet shape, the color was dark or light green, and the smell was normal. The droppings in MMD were unshaped, slightly sticky, and foul smelling. The droppings in SMD were completely unformed, thin-paste-like, and extremely foul smelling. Sampling procedure was as follows: on the evening of the first day of the experiment, each enclosure was thoroughly cleaned and fresh fecal samples of specific individuals were collected at dawn on the second day. Disposable sterile gloves were worn for sample collection to avoid human pollution. Samples were placed in sterile centrifuge tubes immediately after the collection and sealed to avoid cross-contamination between samples. All fresh feces samples were stored in liquid nitrogen and then transported to the laboratory in a mobile refrigerator and frozen at -80°C until the extraction of DNA.

### DNA Extraction, 16S-rRNA Gene Polymerase Chain Reaction (PCR) Amplification, and Sequencing

Total bacterial DNA was extracted using the PowerSoil DNA Isolation Kit (MO BIO Laboratories, Carlsbad, CA, United States) according to the manufacturer’s protocol. The quality and concentration of the extracted DNA were measured using a NanoDrop spectrophotometer (ND-1000, NanoDrop Technologies, Wilmington, DE, United States). The V3–V4 region of the bacterial 16S- rRNA gene was amplified by PCR (95°C for 5 min, followed by 25 cycles of 95°C for 30 s, 50°C for 30 s, 72°C for 40 s, and 72°C for 7 min) using the primers 338F (5′-ACTCCTACGGGAGGCAGCA-3′) and 806R (5′-GGACTACHVGGGTWTCTAAT-3′). Indexed adapters were added to the ends of the primers. The PCR products were mixed with the same volume of 2× loading buffer and were subjected to 1.8% agarose gel electrophoresis for detection. Samples with a bright main band of approximately 450 bp were chosen and mixed in equidensity ratios. Then, the mixture of PCR products was purified using a GeneJET Gel Extraction Kit (Thermo Fisher Scientific, Waltham, MA, United States). Sequencing libraries were validated using an Agilent 2100 Bioanalyzer (Agilent Technologies, Palo Alto, CA, United States) and quantified with a Qubit 2.0 Fluorometer (Thermo Fisher). Finally, paired-end sequencing was conducted using an Illumina HiSeq 2500 platform (Illumina, Inc., San Diego, CA, United States) at Biomarker Bioinformatics Technology, Co., Ltd. (Beijing, China).

### Statistical and Bioinformatics Analyses

The overlapping regions between the paired-end reads were merged using FLASH v1.2.7 and raw reads were quality filtered under specific filtering conditions to obtain high-quality clean tags on the basis of the QIIME (V1.7.0) quality control process. Sequences that were less than 200 bp in length or that contained homopolymers longer than 8 bp were removed. The chimera sequences were detected by comparing tags with the reference database (RDP Gold database) using the UCHIME algorithm and then removed. The effective sequences were then used in the final analysis.

Sequences were grouped into operational taxonomic units (OTUs) using the clustering program UCLUST (version 1.2.22) (Edgar, 2010) against the SILVA bacterial database (Quast et al., 2013) pre-clustered at 97% sequence identity. They were then taxonomically classified to different levels (phylum, class, order, family, genus, and species) using the Ribosomal Database Program (RDP) classifier.

Alpha diversity indices (i.e., ACE, Chao1, Shannon, and Simpson) were calculated by QIIME from rarefied samples using for richness and diversity indices of the bacterial community. Beta diversity was calculated using unweighted UniFrac and non-metric multidimensional scaling (NMDS), after which Intra-group and Inter-group beta distance boxplot diagrams were generated. A one-way analysis of similarity (ANOSIM) was performed to determine the differences in bacterial communities among groups (Clarke and Gorley, 2006). Linear discriminant analysis (LDA) effect size (LefSe) analysis was performed to reveal the significant ranking of abundant modules in healthy and diarrheic samples (Segata et al., 2011). A size-effect threshold of 4.0 on the logarithmic LDA score was used for discriminative functional biomarkers. Phylogenetic Investigation of Communities by Reconstruction of Unobserved States (PICRUSt) (Langille et al., 2013) was used to predict the functional gene content in the fecal microbiota based on taxonomy obtained from the Greengenes reference database^1^ (DeSantis et al., 2006). PICRUSt and LefSe were performed online in the Galaxy workflow framework^2^.

Alpha diversity indexes are presented as the means ± SD. The differences in Alpha diversity indexes and top 10 phyla and genera relative abundances between groups were calculated by use of the Independent-sample *t*-test (for the normally distributed data) or Mann–Whitney *U*-test (for the non-normally distributed data). A *P*-value < 0.05 was considered statistically significant, and *P*-value < 0.01 indicating the differences are extremely significant. The raw sequences obtained in this study have been submitted to the NCBI Sequence Read Archive (accession number SRR6148299).

## Results

### Analysis of 16S-rRNA Gene Sequencing Results

**FIGURE 1:**
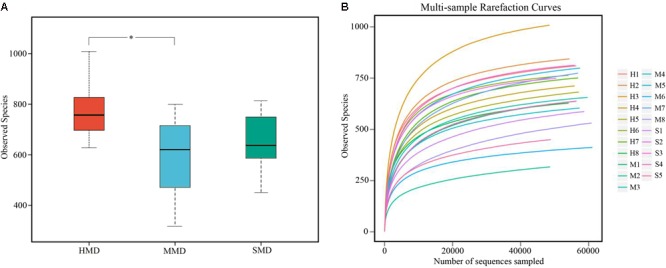
Boxplot and rarefaction curves of operational taxonomic units (OTUs). **(A)** Boxplot of OTUs of observed species. The x-axis shows the observed species (OTUs) and the y-axis shows different groups. The OTU similarity threshold of 97% was considered. Boxes represent the interquartile range (IQR) between the first and third quartiles (25th and 75th percentiles, respectively), and the horizontal line inside the box defines the median. Whiskers represent the lowest and highest values within 1.5 times the IQR from the first and third quartiles, respectively. ^∗^*P* < 0.05 (Student’s *t*-test). **(B)** The rarefaction curves of OTUs. The x-axis shows the number of valid sequences per sample and the y-axis shows the observed species (OTUs). Each curve in the graph represents a different sample and is shown in a different color. As the sequencing depth increased, the number of OTUs also increased. Eventually the curves began to plateau, indicating that as the number of extracted sequences increased, the number of OTUs detected was decreased.

A total of 1,165,731 high-quality sequences were acquired from 8 HMD, 8 MMD, and 5 SMD fecal samples, and 48,489–61,048 (Mean 55,511 ± 3708) analyzed sequences (Mean length = 406.3 bp) were obtained from each sample. A total of 1224 OTUs were obtained at a sequence-similarity level of 97%, with 675 ± 160 (range: 317–1008) as the mean number of OTUs per sample. The observed species (OTUs) in HMD, MMD, and SMD are shown in **Figure 1A**.

The rarefaction curves (Wang et al., 2012) for the OTUs detected in this study showed that the quantity of observed species increased as the sequencing depth increased. The ends of the rarefaction curves tapered off with increasing numbers of sequences per sample, as is commonly observed with sequencing data (**Figure 1B**). Post-filtering sequencing data are displayed in Supplementary Table S1 and the effective sequence-length distribution in all samples shown in Supplementary Figure S1. Good’s Coverage indicates the literature database coverage rate of each sample, and its value in this study was close to 99%, indicating that the majority of the bacteria types in the samples had been detected. Classification of the OTUs using the ribosome database resulted in the detected bacteria being classified into 16 phyla, 26 classes, 43 orders, 63 families, and 160 genera.

### Differences of Microbiota Diversity between Healthy and Diarrheic Forest Musk Deer

**FIGURE 2:**
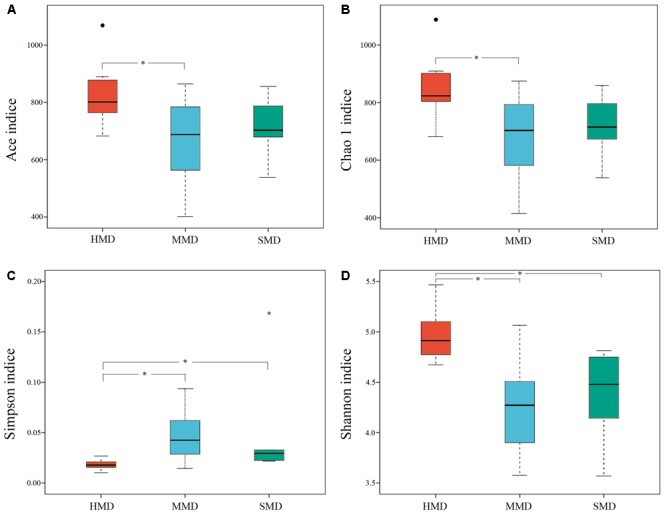
Boxplot of Alpha-diversity indices. Alpha diversity indexes are composite indexes reflecting abundance and consistency. **(A)** Ace and **(B)** Chao1 indices reflect the OTU abundance in samples. **(C)** Shannon and **(D)** Simpson indices reflect the diversity of OTU in samples. The greater the Chao or ACE index, the higher the expected species richness of the microbiota; the smaller the Simpson index, the higher the diversity of the microbiota, and the greater the Shannon index, the higher the diversity of the microbiota. Boxes represent the interquartile range (IQR) between the first and third quartiles (25th and 75th percentiles, respectively), and the horizontal line inside the box defines the median. Whiskers represent the lowest and highest values within 1.5 times the IQR from the first and third quartiles, respectively. “^∙^” Indicates greater than 1.5 times and less than three times the IQR; “^∗^”indicates greater than three times the IQR; ^∗^*P* < 0.05 (Student’s *t*-test).

**FIGURE 3:**
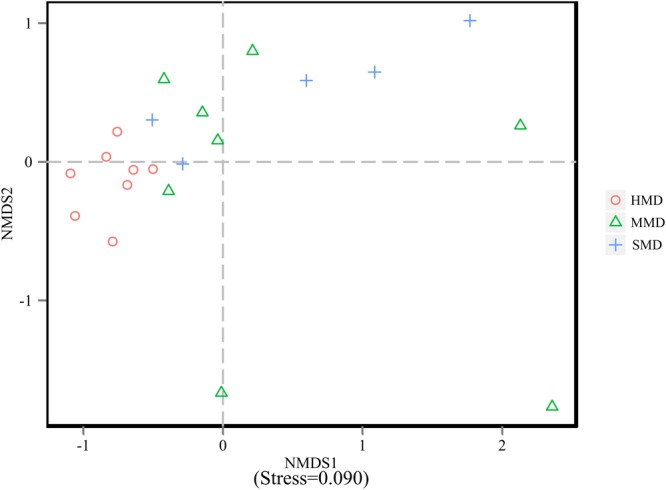
Non-metric multidimensional scaling (NMDS) analysis. Each point in the graph represents one sample, and different colors represent different groups. The distance between points represents the level of difference. Stress lower than 0.2 indicates that the NMDS analysis is reliable. The closer the samples in the graph, the higher their similarity.

**FIGURE 4:**
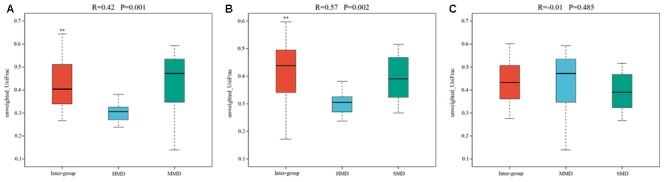
Box plot of Inter-group and Intra-group Beta distance (ANOSIM Analysis). **(A)** Beta distance of HMD and MMD. **(B)** Beta distance of HMD and SMD. **(C)** Beta distance of MMD and SMD. The x-axis represents the grouping and the y-axis represents the distance calculated by Unweighted_unifrac. The data in the box is the distance of Inter-group and Intra-group, respectively. *R*-value: *R*-value range (–1, 1). The *R*-value ≤ 0 represents no significant differences of inter-group and intra-group, and *R*-value > 0 shows that inter-group differences are greater than intra-group differences. *P*-value: the *P*-value represents the confidence level of the statistical analysis; ^∗∗^*P* < 0.01 reflects extremely significant differences between Inter-group and Intra-group. Boxes represent the interquartile range (IQR) between the first and third quartiles (25th and 75th percentiles, respectively), and the horizontal line inside the box defines the median. Whiskers represent the lowest and highest values within 1.5 times the IQR from the first and third quartiles, respectively.

Alpha-diversity indices (Ace, Chao 1, Simpson, and Shannon) (**Figure 2**) and beta-diversity indices of healthy and diarrheic forest musk deer feces were calculated. Comparison between HMD and MMD showed significant differences in Ace (829.80 ± 115.78, 666.89 ± 165.39), Chao 1 (853.39 ± 117.27, 680.73 ± 162.08), Simpson (0.02 ± 0.01, 0.05 ± 0.03), and Shannon (4.96 ± 0.26, 4.25 ± 0.47) indices (*P* < 0.05). Comparison between MMD and SMD showed no significant difference in Ace (666.89 ± 165.39, 712.70 ± 120.20), Chao 1 (680.73 ± 162.08, 716.30 ± 122.31), Simpson (0.05 ± 0.03, 0.06 ± 0.06), or Shannon (4.25 ± 0.47, 4.35 ± 0.51) indices (*P* < 0.05). Comparison between HMD and SMD found significant differences in Simpson (0.02 ± 0.01, 0.06 ± 0.06) and Shannon (4.96 ± 0.26, 4.35 ± 0.51) indices (*P* < 0.05). Beta diversity is used to analyze the temporal and spatial changes in species composition, reflecting whether there is difference in bacterial communities between groups. The NMDS plot showing the dissimilarity of microbial community and also revealed a distinct structure between diarrheic and healthy musk deer (**Figure 3**). Inter-group and Intra-group Beta distance was shown in box plot (**Figure 4**), with the results showing extremely significant differences in bacterial communities between HMD and MMD groups (**Figure 4A**), and between HMD and SMD groups (**Figure 4B**) (*P* < 0.01).

### Relative Abundance and Core Microbiota

**FIGURE 5:**
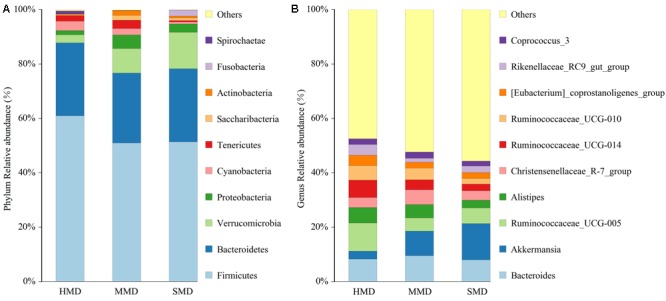
Histogram of relative abundance. The x-axis represents groups and the y-axis represents relative abundance presented as percentage. **(A)** Relative abundance of the top 10 phyla. **(B)** Relative abundance of the top 10 genera. Only the top 10 species in the abundance are shown in the figure; other species were combined as “Others.”

**FIGURE 6:**
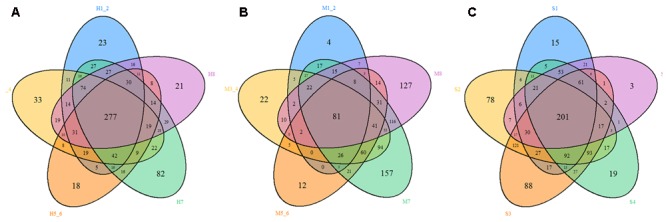
Venn diagram. The Venn diagrams show the numbers of OTUs (97% sequence identity) that were shared or not shared by HMD, MMD, and SMD individuals, respectively, depending on overlaps. For this presentation, two individuals had to be combined (e.g., H1_2) thereby reflecting the number of OTUs shared by both individuals. **(A)** The number of OTUs shared by HMD. **(B)** The number of OTUs shared by MMD. **(C)** The number of OTUs shared by SMD.

The top 10 phyla and the top 10 genera according to relative abundance of the fecal bacteria that were present in HMD, MMD, and SMD samples are displayed in **Figure 5**. At the level of phylum, the dominant bacteria in the HMD, MMD, and SMD were *Firmicutes* and *Bacteroidetes*, followed by *Verrucomicrobia* and *Proteobacteria* (**Figure 5A**). These bacteria accounted for 92.33, 90.73, and 94.71% of the detectable reads in the HMD, MMD, and SMD samples, respectively. The phyla that showed significant differences between HMD and MMD among the top 10 phyla in relative abundance were *Firmicutes, Actinobacteria*, and Spirochaetae (*P* < 0.05). The phyla with significant differences in relative abundance between HMD and SMD were *Firmicutes, Cyanobacteria, Actinobacteria*, and *Fusobacteria* (*P* < 0.05). The phylum with significant difference in relative abundance between MMD and SMD was *Fusobacteria* (*P* < 0.05). On genus level, *Bacteroidetes, Akkermansia*, Ruminococcaceae_UCG-005, and *Alistipes* were the dominant genera (**Figure 5B**). In the top 10 genera in relative abundance, the genera that showed significant differences between HMD and MMD were Ruminococcaceae_UCG-005 and Rikenellaceae_RC9_gut_group (*P* < 0.05). The genera with significant differences in relative abundance between HMD and SMD were Ruminococcaceae_UCG-005, Ruminococcaceae_UCG-014, and Ruminococcaceae_UCG-010 (*P* < 0.05). The shared taxa by all individuals in each group were deemed to represent core bacterial communities. The number of OTUs shared by all individuals within each group was 277, 81, and 201 in the HMD (**Figure 6A**), MMD (**Figure 6B**), and SMD (**Figure 6C**), respectively.

### Significant Difference Analysis and Functional Gene Prediction between Healthy and Diarrheic Forest Musk Deer

**FIGURE 7:**
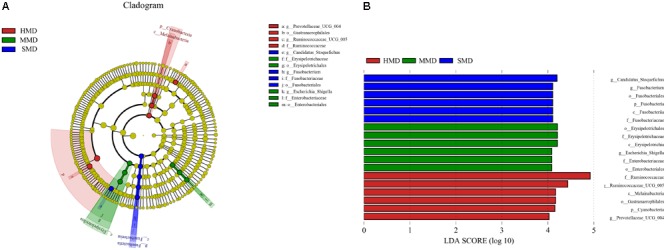
LefSe analysis. **(A)** The cladogram diagram shows the microbial species with significant differences in the three groups. Red, green, and blue indicate different groups, with the species classification at the level of phylum, class, order, family, and genus shown from the inside to the outside. The red, green, and blue nodes in the phylogenetic tree represent microbial species that play an important role in the HMD, MMD, and SMD groups, respectively. Yellow nodes represent species with no significant difference. **(B)** Species with significant difference that have an LDA score greater than the estimated value; the default score is 4.0. The length of the histogram represents the LDA score; i.e., the degree of influence of species with significant difference between different groups.

LefSe analysis was performed to reveal the significant ranking of abundant modules. The cladogram (**Figure 7A**) showed differences in 18 taxa among HMD, MMD, and SMD. The plot from LefSe analysis (**Figure 7B**) displays LDA scores of microbial taxa with significant differences among HMD, MMD, and SMD. On genus level, the biomarker demonstrating significant differences between the HMD group and the other two groups were Prevotellaceae_UCG-004 and Ruminococcaceae_UCG-005; the biomarker showing significant differences between the MMD group and the other two groups was *Escherichia–Shigella*. In addition, the biomarker showing significant differences between the SMD group and the other two groups were *Fusobacterium* and Candidatus_Stoquefichus. Relative abundance of *Escherichia–Shigella* and *Fusobacterium* between the three groups was displayed in Supplementary Figure S2.

**FIGURE 8:**
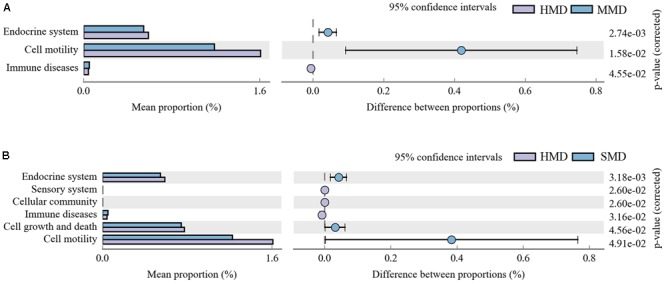
**(A)** The abundance ratio of different functions between HMD and MMD. **(B)** The abundance ratio of different functions between HMD and SMD. PICRUST analysis. Variance analysis of the KEGG metabolic pathways in the second level. The graphs show the abundance ratio of different functions in two groups of samples. The middle shows the difference between proportions of functional abundance in the 95% confidence interval, and the value at the rightmost is the *P*-value. *P* < 0.05 represents the significant difference.

The species composition information obtained by comparing 16S sequencing data via PICRUSt software was used to infer the functional gene composition in samples. By variance analysis of Kyoto Encyclopedia of Genes and Genomes (KEGG^3^) metabolic pathways, the differences and changes of metabolic pathways of functional genes in microbiota between the samples of different groups could be observed. Comparing the HMD and MMD groups, a total of three gene metabolic pathways showed significant differences (*P* < 0.05) (**Figure 8A**). Comparing the HMD and the SMD groups, a total of six gene function metabolic pathways showed significant differences (*P* < 0.05) (**Figure 8B**). There was no significant difference in the metabolic pathways between the MMD and SMD groups (*P* > 0.05).

## Discussion

In this study, 16S-rRNA gene Illumina HiSeq sequencing was used to study the intestinal microbiota in healthy captive-bred forest musk deer, and in deer from the same facility with mild and severe diarrhea. The results showed that the intestinal microbiota in healthy musk deer exhibited high diversity and rich bacterial species content, whereas the diversity of musk deer with diarrhea is significantly reduced, which is similar to previous observations in humans (Frank et al., 2007), dogs (Minamoto et al., 2015), and horses (Schoster et al., 2017) with diarrhea. Therefore, bacterial species richness and diversity are considered to represent important components of a “healthy” intestinal microbiome. This is consistent with their important role in maintaining the dynamic balance of the intestinal micro-ecosystem and ensuring normal physiological functions.

The histogram of relative phylum abundance showed that regardless of the presence of diarrhea, *Firmicutes* and *Bacteroidetes* comprised the main dominant bacteria. However, the relative abundance of *Firmicutes* in the feces of healthy forest musk deer was significantly higher than that of musk deer with diarrhea. Notably, *Firmicutes* constitutes the main type of bacteria that catabolize cellulose in food and degrade cellulose into volatile fatty acids for the host (Sundset et al., 2007; Gruninger et al., 2014; Ishaq and Wright, 2014). In addition, *Firmicutes* can also regulate the immune response *in vivo*, inhibiting the invasion of opportunistic pathogens and preventing intestinal inflammation (Atarashi et al., 2011; Zhang et al., 2015). The reduction of *Firmicutes* abundance in the intestinal tract of musk deer with diarrhea would likely concomitantly reduce the digestive physiological functions of the species. The results obtained here are consistent with the results of research on horses with diarrhea by Costa et al. (2012). Furthermore, the comparison of beta diversity showed extremely significant differences of bacterial communities between healthy and diarrhea forest musk deer, which was correlated with the abnormal digestive physiological functions of musk deer.

LefSe analysis showed that on genus level, the biomarker demonstrating significant difference between the MMD group and the HMD and SMD groups constitute *Escherichia–Shigella*. *Escherichia–Shigella* is common pathogens, the main transmission paths of which comprise the feces of the infected subject, food, and water containing the bacteria. The pathogen can cause inflammation of the colon mucosa, resulting in symptoms such as abdominal pain, diarrhea, and mucosanguineous stool (Ogunsanya et al., 1990). Therefore, food and water for feeding the musk deer and manure management are indicated as keys to reducing mild diarrhea. In contrast, the biomarker showing significant differences between the SMD group and HMD and MMD groups constitute *Fusobacterium*. *Fusobacterium* is considered to be an inflammatory microbe and biomarker that can inhibit T cell responses *in vivo* and promote the expression of inflammatory factors (Nosho et al., 2016; Wei Z. et al., 2016). The relative abundance of *Fusobacterium* is also found to be increased in horses with colitis (Costa et al., 2012) and in humans with diarrhea (The et al., 2017). Comparing the MMD group with the SMD group, it may be speculated that *Fusobacterium* may proliferate in a background of mild diarrhea and become a dominant pathogen. Thus, the physiological disorder of the intestine in forest musk deer is correlated with the existence of a major pathogen type.

PICRUSt analysis indicated that in comparison with the HMD group, the abundance of functional genes in the metabolic pathways of immune diseases significantly increased in forest musk deer with diarrhea. The presence of intestinal microbiota can stimulate the development of the host immune system, enhance host immune function (Oikonomou et al., 2013) and prohibit the invasion of pathogenic microbes (Slifierz et al., 2015), whereas diarrhea caused by an intestinal microbiota disorder can affect the host’s immune capacity, leading to increased susceptibility to immune diseases. In addition, it may also have an impact on cellular processes and metabolic pathways of organismal systems in the host. However, PICRUSt is only a means of predicting functional genes; thus, further research is required to confirm the accuracy of gene function information by metagenomic analysis.

The structure and composition of intestinal microbiota in diarrheic forest musk deer exhibited more complexity with greater fluctuation than those in healthy musk deer, suggesting that there may be many underlying reasons for diarrhea in musk deer. Forest musk deer are naturally wary and timid, have strong physiological stress responses, and represent typical neurotic animals (He et al., 2014). Therefore, we believe that some cases of diarrhea may not be derived from pathogens but rather from irritable bowel syndrome (IBS). The occurrence of IBS is affected by the gut-brain neural circuit and is usually associated with mental factors; in particular, emotional stress, anxiety, and fear can cause disorders of gastrointestinal contraction and dilation as well as of secretion functions (Ohman and Simrén, 2010). Studies indicate that human patients with IBS also exhibit intestinal microbiota imbalance, although whether this change in intestinal microbiota is primary or secondary is not yet clear (Mättö et al., 2005). Our experience suggests that stress responses triggered by disturbing stimuli may lead to alterations of intestinal microbiota, which further results in pathological diarrhea and even severe diarrhea; i.e., Physiological stress may constitute the initial trigger for diarrhea. However, this association has yet to be confirmed. Consequently, to avoid diarrhea, captivities should take special care with conditions that could lead these animals to stress. In addition, the occurrence of diarrhea may also have a viral cause, such as Bovine viral diarrhea virus (BVDV). It has been reported that BVDV does not possess strict host-specificity and infections of over 50 species in the mammalian (Passler et al., 2016; Graham et al., 2017). But the 16S-rRNA gene sequencing can’t detect viruses. Therefore, metagenomics or a viral microarray research is essential in the future.

So far, forest musk deer breeding in China is mainly distributed in two regions: Qinling mountainous area in southern Shaanxi Province, and western mountainous area in Sichuan Province. The farming deer in these two regions account for 90% of captive musk deer populations in China. Of them, Shaanxi Province holds 70% of captive deer population from the two provinces. All forest musk deer farming in Shaanxi are very similar in altitude, climate, fodder types, breeding patterns, and so on. The diseases of the captive deer in Qinling mountainous area in southern Shaanxi Province is almost the same. Therefore, the results from this research (Pien Tze Huang Forest Musk Deer Breeding Center, Shaanxi) can represent the situation in the whole area of Qinling Mountains, Shaanxi, but it’s not sure whether our findings could be extended to western part of Sichuan, and further confirmation is needed.

Historically, forest musk deer farms have generally used antibiotics to treat individuals with diarrhea. However, the blind use of antibiotics may eliminate sensitive beneficial bacteria that act as protective barriers and prohibit foreign bacterial invasion, aggravate microbiota imbalance, and even cause fungal infection to aggravate diarrhea (Edlund and Nord, 2000; Donskey et al., 2003; Johnston et al., 2007). Therefore, in order to prevent further aggravation of the microbiota disorder as identified in the current study, appropriate use of probiotics should be considered for adjuvant therapy. In summary, this study provides a theoretical basis for exploration of the treatment of diarrhea in forest musk deer, and has guiding value for improving the health and potential breeding of forest musk deer.

## Author Contributions

YL and DH conceived and designed the experiments. YL, XH, SY, and JZ carried out the DNA extraction and data analysis. MF, XS, SX, and MC participated in the sample collection. YL and ShuL wrote the paper. XH, LQ, MZ, and ShaL assisted with experiments and advised on manuscript content. All authors read and approved the final manuscript.

## Conflict of Interest Statement

The authors declare that the research was conducted in the absence of any commercial or financial relationships that could be construed as a potential conflict of interest.

## Footnotes

1 http://greengenes.secondgenome.com/

2 https://huttenhower.sph.harvard.edu/galaxy/

3 http://www.genome.jp/kegg/kegg2.html
