# Supplementary material for: Comparison Between the Fecal Bacterial Microbiota of Healthy and Diarrheic Captive Musk Deer
Source: Front Microbiol. 2018 Mar 2;9:300. doi: 10.3389/fmicb.2018.00300 (PMC5840195; doi:10.3389/fmicb.2018.00300)
Supplement: Supplementary file 1 [file Data_Sheet_1.DOCX]

Supplementary Material

**Comparison between the Fecal Bacterial Microbiota of Healthy and Diarrheic Captive Musk Deer**

**Yimeng Li^1^, Xiaolong Hu^2^, Shuang Yang^1^, Juntong Zhou^1^, Lei Qi^1^, Xiaoning Sun^1^, Mengyuan Fan^1^, Shanghua Xu^1^, Muha Cha^1^, Meishan Zhang^1^, Shaobi Lin ^3^, Shuqiang Liu*^,1, 3^, Defu Hu*^,1^**

*****Corresponding authors: Shuqiang Liu, [liushuqiang@bjfu.edu.cn](mailto:liushuqiang@bjfu.edu.cn); Defu Hu, hudf@bjfu.edu.cn

1. Supplementary data

2. Supplementary Figures and Tables

| Sample | PE_reads | Nochimera | AvgLen(bp) | GC(%) | Effective(%) |
| --- | --- | --- | --- | --- | --- |
| HMD1 | 79922 | 67311 | 411 | 52.45 | 84.22 |
| HMD2 | 79914 | 66344 | 414 | 51.84 | 83.02 |
| HMD3 | 79857 | 65191 | 413 | 52.38 | 81.63 |
| HMD4 | 80161 | 66439 | 411 | 52.32 | 82.88 |
| HMD5 | 79797 | 66914 | 410 | 52.58 | 83.86 |
| HMD6 | 79981 | 66253 | 413 | 52.29 | 82.84 |
| HMD7 | 80366 | 66738 | 414 | 51.55 | 83.04 |
| HMD8 | 80226 | 66524 | 412 | 52.35 | 82.92 |
| MMD1 | 79986 | 63571 | 413 | 52.08 | 79.48 |
| MMD2 | 64897 | 53160 | 413 | 54.34 | 81.91 |
| MMD3 | 80099 | 66231 | 412 | 52.37 | 82.69 |
| MMD4 | 80110 | 64612 | 415 | 52.23 | 80.65 |
| MMD5 | 80115 | 66831 | 412 | 52.21 | 83.42 |
| MMD6 | 80079 | 66343 | 417 | 49.92 | 82.85 |
| MMD7 | 80035 | 64290 | 413 | 52.39 | 80.33 |
| MMD8 | 80130 | 66440 | 414 | 52.03 | 82.92 |
| SMD1 | 80005 | 65704 | 415 | 53.02 | 82.12 |
| SMD2 | 71890 | 58907 | 414 | 52.11 | 81.94 |
| SMD3 | 80012 | 65798 | 410 | 52.97 | 82.24 |
| SMD4 | 75442 | 61679 | 413 | 53.49 | 81.76 |
| SMD5 | 64620 | 52761 | 415 | 52.54 | 81.65 |

**Supplementary Table S1. Statistical table of post-filtering sequencing data.** Sample: Name of sequencing sample (HMD: healthy musk deer; MMD: moderate diarrhea musk deer; SMD: severe diarrhea musk deer); Paired-End (PE) reads: Number of original PE reads; Nochimera: Number of valid sequences after removal of chimeras; AveLen (bp): Average length of valid sequences; GC (%): GC content of valid data; Effective (%): The percentage of valid sequences after chimera removal over the original number of PE reads.


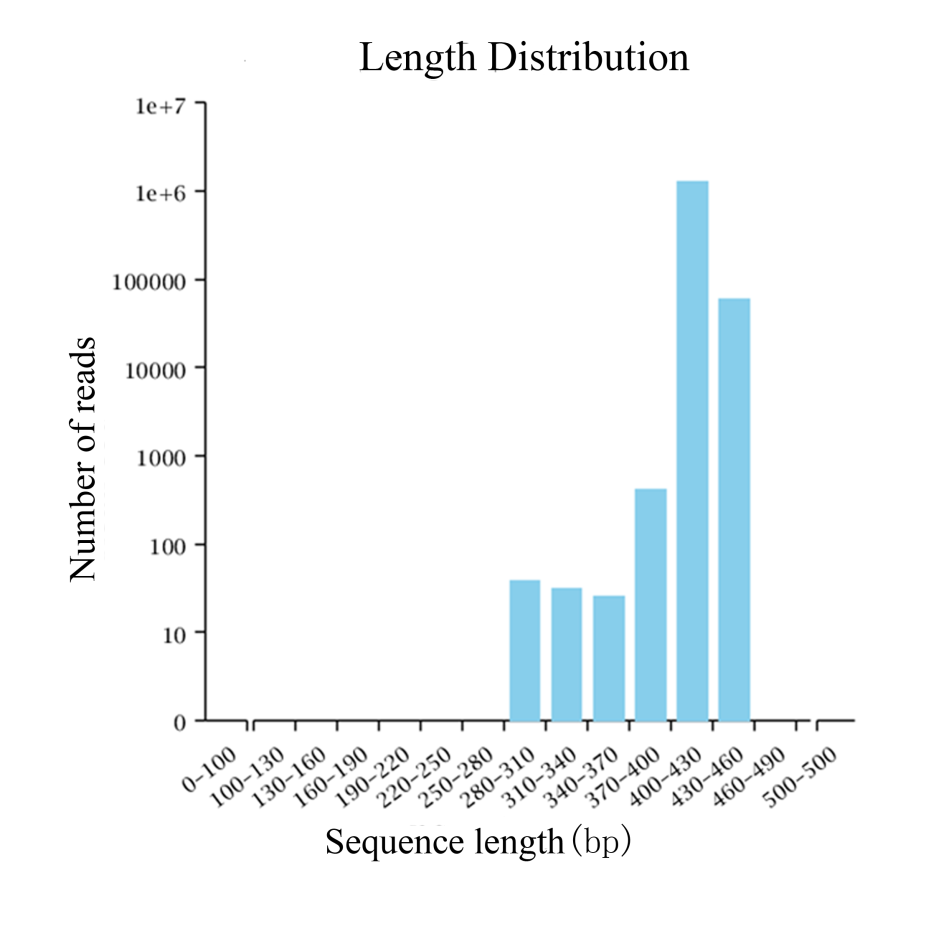


**Supplementary Figure S1. Effective sequence-length distribution.** The X-axis shows the sequence length (bp) and the Y-axis shows the number of reads with each different length (Number of reads).


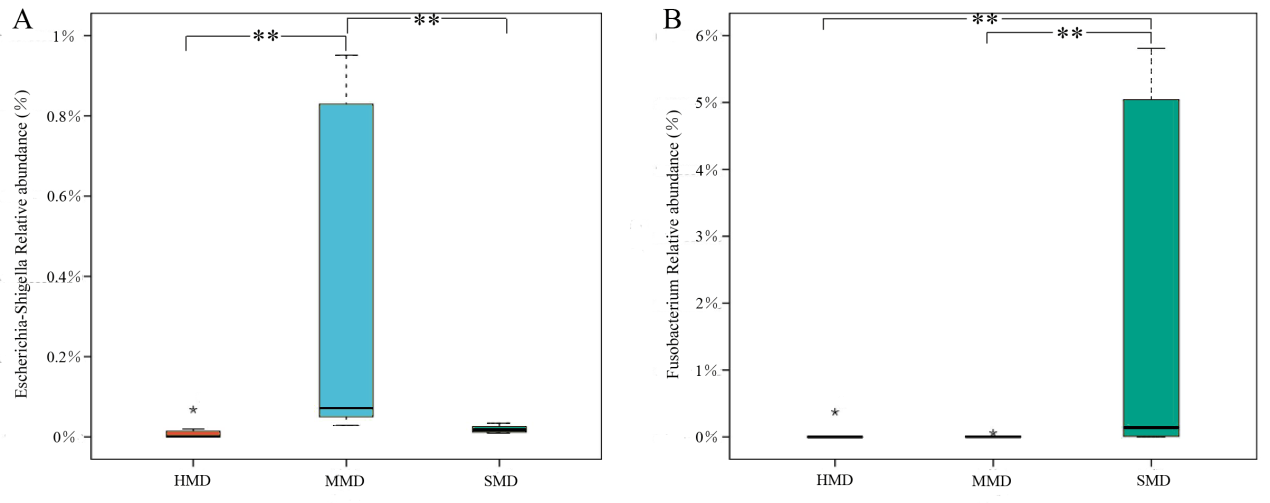


**Supplementary Figure S2. Box plot of relative abundance.** The x-axis represents groups and the y-axis represents relative abundance presented as percentage. (A) Relative abundance of Escherichia-Shigella. (B) Relative abundance of Fusobacterium. Boxes represent the interquartile range (IQR) between the first and third quartiles (25th and 75th percentiles, respectively), and the horizontal line inside the box defines the median. Whiskers represent the lowest and highest values within 1.5 times the IQR from the first and third quartiles, respectively. **P < 0.01 (Student’s t-test).
